# Integration of urban science and urban climate adaptation research: opportunities to advance climate action

**DOI:** 10.1038/s42949-023-00113-0

**Published:** 2023-06-10

**Authors:** José Lobo, Rimjhim M. Aggarwal, Marina Alberti, Melissa Allen-Dumas, Luís M. A. Bettencourt, Christopher Boone, Christa Brelsford, Vanesa Castán Broto, Hallie Eakin, Sharmistha Bagchi-Sen, Sara Meerow, Celine D’Cruz, Aromar Revi, Debra C. Roberts, Michael E. Smith, Abigail York, Tao Lin, Xuemei Bai, William Solecki, Diane Pataki, Luís Bojorquez Tapia, Marcy Rockman, Marc Wolfram, Peter Schlosser, Nicolas Gauthier

**Affiliations:** 1 School of Sustainability, College of Global Futures, Arizona State University, Tempe, Arizona USA; 2 Urban Ecology Research Laboratory, Department of Urban Design and Planning, University of Washington, Seattle, Washington USA; 3 Climate Change Science Institute, Oak Ridge National Laboratory, Oak Ridge, Tennessee USA; 4 Mansueto Institute for Urban Innovation, University of Chicago, Chicago, Illinois USA; 5 Human Dynamics Section, Oak Ridge National Laboratory, Oak Ridge, Tennessee USA; 6 Urban Institute, University of Sheffield, Sheffield, United Kingdom; 7 School of Geographical Sciences and Urban Planning, Arizona State University, Tempe, Arizona USA; 8 International Centre for Climate Change and Development, Dhaka, Bangladesh; 9 Indian Institute for Human Settlements, Bangalore, India; 10 School of Life Sciences, University of KwaZulu-Natal; Sustainable and Resilient City Initiatives Unit, EThekwini Municipality, Durban, Republic of South Africa; 11 School of Human Evolution and Social Change, Arizona State University, Tempe, Arizona USA; 12 Institute of Urban Environment, Chinese Academy of Sciences, Xiamen, People’s Republic of China; 13 Fenner School of Environment & Society, Australian National University, Canberra, Australia; 14 Institute for Sustainable Cities, Hunter College, City University of New York, New York, New York, USA; 15 Laboratorio Nacional de Ciencias de la Sostenibilidad, Instituto de Ecología, National Autonomous University of Mexico, Mexico D.F., Mexico; 16 Department of Anthropology, University of Maryland, College Park, Maryland USA; 17 Leibniz Institute of Ecological Urban and Regional Development, Dresden, Federal Republic of Germany; 18 Julie Ann Wrigley Global Futures Laboratory, Arizona State University, Tempe, Arizona USA; 19 Florida Museum of Natural History, University of Florida, Gainesville, Florida USA

**Keywords:** Geography, Science, technology and society

## Abstract

There is a growing recognition that responding to climate change necessitates urban adaptation. We sketch a transdisciplinary research effort, arguing that actionable research on urban adaptation needs to recognize the nature of cities as social networks embedded in physical space. Given the pace, scale and socioeconomic outcomes of urbanization in the Global South, the specificities and history of its cities must be central to the study of how well-known agglomeration effects can facilitate adaptation. The proposed effort calls for the co-creation of knowledge involving scientists and stakeholders, especially those historically excluded from the design and implementation of urban development policies.

## Introduction

Urban areas and urbanized regions present concentrations of populations vulnerable to the consequences of climate change but also have significant potential to reduce societal vulnerability through an enhanced adaptive capacity to mitigate its impacts^1^. The principal challenge is how to minimize growing vulnerabilities while enabling far-reaching and equitable climate action for sustained and sustainable development. Urban-focused climate adaptation research is central to this discussion. Urbanization tends to refer to the population shift from rural to urban areas, a perspective that presupposes a clear distinction between urban and rural. Here we use the term urbanization to refer to the agglomeration of population settlements of diverse types, scale and density. What is seen as essential in the urbanization process is the concentration of populations thereby increasing proximity and closeness in physical space which in turn facilitates interactions in social space.

According to the IPCC^2^, urbanization offers a global and time-limited opportunity to work towards widespread and transformational adaptation and climate-resilient development. Local governments have taken the lead—ahead of national governments—in developing comprehensive adaptation plans^3^. These plans are increasingly aligned with international agreements, such as the Sendai Protocol, the UN Sustainable Development Goals, the Paris Climate Change Agreement, and the New Urban Agenda. Recent research and data analyses have indeed emphasized the disproportionate importance of cities and urbanization in achieving the goals set by these international agreements^4^. Nevertheless, only a few of these plans have been fully implemented, and the urban share of global GHG emissions continues to increase^5^. Furthermore, it has become increasingly clear that many proposed climate adaptation plans can exacerbate urban poverty, promote gentrification, and aggravate long-standing environmental injustices^6^. Therefore, the scientific and practice communities propose ongoing dialogues to link climate action and development in a coherent set of transitions framed by climate-resilient, environmentally sustainable and equitable socioeconomic development action plans^7^.

Climate adaptation requires social interventions and resource investments which are especially challenging against a backdrop of inflation, rising costs of living, energy poverty and economic insecurity affecting not only low-income countries. Urgent problems such as unemployment and underemployment, entrenched poverty, lack of affordable housing, and inadequate access to public services demand policy attention. While these challenges are exacerbated by climate vulnerabilities, adaptation as a practice has become increasingly siloed into technocratic and managerial mindsets, characterized by hyperlocal and fragmented approaches, distinct from the broader scope and scale of transformative action that it requires^8^. To inform effective and equitable actions, we argue for the development of a concerted research effort on urban-focused climate adaptation. We recognize that the concept of climate change adaptation, although widely used, is often fraught with ambiguity and hampered by the lack of agreed-upon definition^9^. Here we define urban adaptation to climate change (hereafter ‘urban adaptation’) as the set of actions by which urban societies adjust, change, and transform their energy systems, economies, infrastructures, support systems, interactions, and governance mechanisms to mitigate the adverse effects on urban communities of the environmental changes brought about by climate change. Urban adaptation does not imply that all components of urban societies and urban systems need to change at the same time. There are temporal and scale differences in the implementation of adaptation solutions, as well as differences in who takes adaptation efforts and who benefits from them. Furthermore, whether a set of actions is useful can change as new information and conditions emerge, especially in a non-stationary climate and technological future. Adaptation is therefore also about facilitating processes that enable actions or responses.

Implementable research on urban adaptation must integrate the social, ecological, and technological interactions which, across time and space, constitute urban systems. We refer to urban systems, rather than cities, to underscore the complex reality that what constitutes the urban defies political and administrative boundaries, crosses spatial scales, and is best understood in terms of dynamic and networked relations. Relevant research questions on urban adaptation have been posed already^10^ but here we put forth a revised set of questions, constituting a research agenda, motivated by the multifaceted nature of cities and urban systems. Some examples of these questions, emerging at the junctures between different disciplinary perspectives, are proposed in Table 1. The proposed research agenda is grounded on eight tenets. (1) Multiple and networked urban actors (individual and collective, formal and informal, public and private) are involved in the socioeconomic development of urban areas. (2) Urban settings concentrate and accelerate interactions and their social, economic and political outcomes in space and time. (3) The historical trajectories of cities result from technological capabilities and socioeconomic processes. (4) Climate risk exposure and adaptive capacity vary with the scale and heterogeneity of urbanization. (5) The vulnerabilities of urban systems should be understood, and adaptive capacities developed, with careful attention to how their history channeled their current conditions. (6) The nexus of climate change, biodiversity, ecosystem services, and urban development must be considered; (7) urban climates are partly socially constructed. (8) Co-creation of knowledge among public and private sectors as well as citizens, specifically the urban poor and residents of informal settlements, must be part of the new research agenda.

**Table 1:** Research questions for a global research agenda on urban adaptation.

Area of investigation	Research questions
Urbanization and exposure of urban areas to climate change	1. How do current urbanization patterns compound climate change exposures? 2. What is the relationship between cascading risks and different models of past, present and future urban and infrastructure development? 3. What types of infrastructure minimize exposure to climate change? How to make urban infrastructures less vulnerable to the effects of climate change? 4. What are the specific risks that emerge in new forms of urbanization and informal settlements? 5. What are the limits―environmental, technological, organizational―to urban adaptation? What conditions make “managed retreat” an effective response to reduce exposure to climate change? What are the trade-offs between “protecting and staying in place” and relocating?
Differential vulnerabilities	6. How do different urban conditions create different patterns of vulnerability to climate change in urban areas? 7. How do social inequities and spatial disparities compound exposure of urban community? 8. How do social inequities and spatial disparities compound exposure of urban community? 9. In which way do people’s life trajectories change their vulnerability to climate risks?
Socio-ecological and technological innovations	10. What socio-ecological interactions enhance adaptation in urban areas? How can these be fostered? 11. What threatens the adaptability of socio-ecological functionalities in urban areas? 12. How can Nature-Based Solutions expand cities’ adaptive capacity? 13. What are the trade-offs between securitizing the city via physical infrastructures or improving the adaptability of socio-ecological systems?
Urbanization and resilient socioeconomic development	14. Which of the generative processes that make urbanization a driver of socioeconomic development are more resilient to climate change? 15. Which of the generative processes that make urbanization a driver of socioeconomic development are more vulnerable to climate change? 16. How can a historical perspective on urbanization and its role in socioeconomic development inform the design of adaptation policies? 17. How can efforts at furthering mitigation and adaptation foster urban socioeconomic development? 18. How can energy-poverty and insufficient mobility services be provided while furthering urban adaptation?
Social policy	19. What social policies are most effective in fostering adaptation? 20. What are the dynamics of relocation, and how to minimize their impacts? 21. In what ways can innovations in insurance provision against the effects of climate change facilitate urban adaptation? 22. How can health care and education systems increase adaptability and reduce vulnerability?
Inclusive governance	23. How can inclusive urban governance systems be developed to deliver adaptation at scale? 24. Is there evidence that inclusive governance facilitates adaptation? 25. How do different governance systems exclude and oppress vulnerable population sectors, hence reducing adaptation? 26. How can the experiences and insights accumulated by the populations of informal settlements and poor urban communities (who have a history of adapting to social and economic dislocations) inform the design of climate change adaptation-enhancing policies?

To achieve global scope and applicability, research on urban adaptation to climate change must confront the challenges of multi-level, multi-actor and polycentric governance of and in urban systems, acknowledge the Global North’s relatively greater share of global greenhouse gas (GHG) emissions, and address the prevalence of urban informality in the Global South and its growing presence in the Global North. Such an effort must also address the inherent inequalities embedded in knowledge production, which favor perspectives from the former colonizing powers and tend to reproduce the socioeconomic systems that produce climate change and drive vulnerabilities to its impacts. Below, we address how novel research in urban adaptation can be developed through transdisciplinary collaborative efforts.

### Urban adaptation research: imperatives and challenges

Cities occupy less than 2% of the land surface but house 65% of the world’s population, with a projected increase of 2.5 billion urban dwellers by 2050^11^. The growth (or decline) of cities unquestionably reshapes overall land use, degrades ecosystems and natural landscapes, and perpetuates the development of underdevelopment/disparities^12,13^. Cities in the Global North―typically growing more slowly, with some even experiencing shrinking populations―approach climate change as a challenge to enhance their resilience and often an opportunity to close equity gaps^14^. By contrast, fast-growing cities, particularly in the Global South, see investments in urban adaptation (e.g., emissions reduction) competing with their urgent need to provide public goods and basic infrastructure and services^15^. The risks posed by extreme weather events and changes in climate patterns are leading policymakers and practitioners worldwide to take steps to confront immediate and expected adverse consequences^16^. Many cities (such as those in the C40 compact and the 100 Resilient Cities) are responding to climate change by creating new kinds of policy approaches in the form of comprehensive climate and sustainability plans addressing both near and long-term impacts^3^.

Cities and their inhabitants have always had to contend with environmental variability^17^. What has changed recently is the pace and increasing severity and variability of extreme weather events which are projected to affect many more urban areas, and thus much larger populations into the foreseeable future. Variability beyond historical climatic envelopes calls for adaptative capacities that go beyond risk management and mitigation. Landscape learning―how humans learn about the environments they inhabit and modify their behavior accordingly―is an important component of climate adaptation^18^. This learning is manifested in ‘urban landscapes’ and is not easy to transform or replace, as attested by the difficulties in decarbonizing the supply of electricity to cities in the US, Germany, India, or China^19^. Conventional adaptation strategies, via hardened infrastructure or the expansion of air conditioning use, are also subject to catastrophic failures, long-term deterioration, and positive feedback that amplify climate change harms. There are natural limits to adaptation as well, such as more slow-changing risks to cities through coastal inundation and sea-level rise^2^.

The ongoing implementation of climate adaptation measures in cities relies on the importance of systemic knowledge that can anticipate new scenarios, harness the positive effects of urbanization, and inform the management of adverse distributional effects and unintended (negative) consequences^20^. Urban adaptations have consequences at many scales from local communities’ health to global geopolitics, biodiversity, and trade^21^. As adaptation practice advances, stakeholders are recognizing the complexity of the decisions involved, as well as the need to remain agile and adaptive to respond to multiple, intersecting, and dynamically cascading risks^22^. Moreover, acts of commission and omission can exacerbate urban injustices^23^; and urban adaptation governance must correct long-standing legacies of resource and risk distribution embedded in the current urban physical and social infrastructure^24^.

### From urban science to research on urban adaptation

Urban adaptation research must contend not only with the contemporary complexities of modern urban forms but also with the history through which they have developed. To advance an impactful research effort on urban adaptation, we need to build upon our current understanding of cities and urban development processes. The accumulation of discipline-specific insights has given rise to an appreciation of the city as a synthetic unit of analysis^25^. Recent advances in digitalization and computation have led to new measurements of a diverse set of properties and behaviors in cities spanning a wide latitude of cultures, levels of development, and geographies. The comparative analysis of this growing body of evidence has reinvigorated the study of cities with a focus on understanding common generative processes while recognizing the importance of local contexts and histories.

The emergent urban science^26–29^ is inter- and transdisciplinary, convergent, and supports an expanded set of quantitative models providing testable expectations about the fundamental aspects of cities and urban activities. Notwithstanding cultural, technological, and political differences across time and space, cities and urban systems share fundamental generative processes, stemming from the concentration, mixing and interaction of populations, and exhibit empirical regularities linking many of their salient characteristics such as population size, areal extent, density, and infrastructure^30,31^. Yet, urban science is not all-encompassing and must be aligned with complementary analysis of urban processes that build on research that emerges from the urban experience with a focus on the co-production of knowledge with study subjects. The co-production approach offers local, fact-based sense-making of lived urban experiences and opportunities for urban development^32,33^. This grounded understanding of cities provides a foundation for novel research in urban adaptation.

Systematic research and practice on urban adaptation worldwide have grown in importance, as evidenced by the attention the IPCC has paid to the role of cities in overall climate change adaptation efforts^34,35^. The challenge is to create a synthetic analytical framework, agreed-upon metrics, shared goals and comparable indicators. Urban space is constituted through frequent and intense social interactions among a diversity of individuals, activities and organizations enabled and supported by physical infrastructure and the distribution of services^36–38^. In high-income nations, urban adaptations tend to focus on the physical aspects of the city given the urgency of protecting urban infrastructure. Examples include discussions on how infrastructure and services (e.g., utilities) as engineered systems can withstand the effects of frequent and intense extreme weather events brought about by climate change^39–41^. Contrasting these concerns are sustainability issues related to growing socioeconomic disparities in cities, especially in the fast-growing cities of the Global South^42^. Equity and development challenges place the focus of urban climate adaptation on the social, economic, and political aspects of cities. Impactful research on urban adaptation demands that we understand the dynamic interplay between the built environment and underlying support systems (e.g., utilities) that are designed to serve human and ecological well-being across spatial scales.

**Fig. 1:**
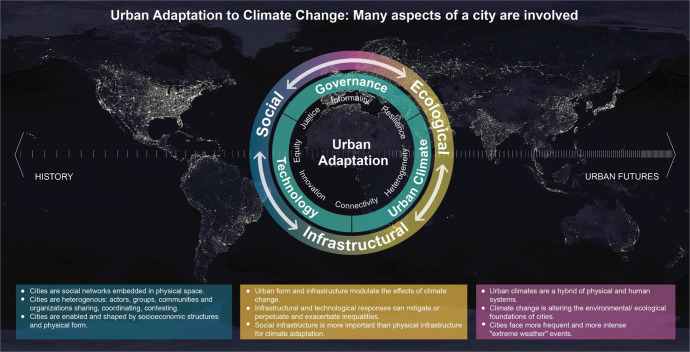
The multifaceted nature of urban adaptation to climate change. The inner circle identifies the main factors constraining urban adaptation. The outer circle refers to the processes facilitating adaptation.

Figure 1 illustrates the multifaceted nature of urban adaptation to climate change. Cities are complex social, ecological, and infrastructural systems. The intersections of urban climate, technology, and governance lead to the large number of interacting components that define the scope and emerging challenges of a convergent global research agenda on urban adaptation. The figure places the research agenda in a historical perspective to bridge the lesson learned from past urbanization to the development of future cities.

### Cities as complex social-infrastructural-ecological networks

As systems of intertwined social, ecological, and technological interactions―connecting individuals, communities, organizations, institutions, activities, technologies, infrastructure and ecosystems―cities comprise networks that exhibit unique properties^43,44^. These networks constitute the unit of analysis for the science of urban adaptation to climate change. The social networks forged by urban dwellers provide channels and relationships through which information is generated and shared^45^. This information regulates the functioning of cities themselves but also orchestrates resource and energy flows worldwide. These information networks are complex networks, i.e., the patterns of connection are neither purely regular nor purely random^46^. Cities facilitate greater interconnectivity between different people and organizations, which in turn is the result of the reduction of physical distance entailed by greater population densities and of supporting services, infrastructure, and institutions^13^. Urbanization also entails complex changes in governance structures and the proliferation of multiple actors able to act in urban environments. The transformation of spatial relations through urbanization and the changing relations between the city, its hinterland and the broader spaces that the city depends upon calls for sophisticated understandings of ways in which the city is governed whether this is because of the need to understand how different levels of government intervene in the city, or the need to understand how multiple actors within and beyond the state (including communities, business, and individuals) are able to deliver purposeful action to address climate change in the city.

The agglomeration of individuals and activities in urban areas has costs and benefits that accrue to both people and the environment. Economically, denser socioeconomic networks promote mechanisms that support economic growth such as specialization, the division of labor, diversity of skills, and the creation and sharing of knowledge^47^. Civic institutions and public services, as well as community organizations and collective action, may enable a fairer (re)distribution of resources and equity. Social conflicts affect collective life in cities, shaping the ability of human societies to adapt, sometimes opening alternative courses of action. For example, higher density is generally considered to facilitate reductions in CO2 emissions. But density also contributes to urban inequality and hence vulnerability to climate change impacts^48^. Costs associated with urban density include congestion, housing, pollution, health, reduced access to greenspaces and personal insecurity. The impact of heatwaves during 2022 in Asia and Europe has further brought into question densification strategies in the context of adaptation. The ongoing COVID-19 pandemic reminded us of the special vulnerability of cities to emerging zoonotic outbreaks^49^, a feature of cities throughout history^50^. It should also be noted that cities have historically shown great resilience in recovering from the devastating effects of plagues^51^. The recent pandemic highlights the paramount importance of cities’ governance capacities to respond to exogenous shocks^52^. A key question for adaptation research is how urban agglomeration can support inclusive economies and social innovation while expanding processes resilient to the myriad effects of climate change.

Cities are extended complex socio-ecological and technological systems^53^. Urban networks extend far beyond their immediate spatial and social domain^54,55^. The structure of these regional and international networks has several general properties, creating a structural hierarchy that channels energy and resource flows from rural areas and smaller places to larger cities, with information (financial, media, technology) flowing primarily in the opposite direction^56^. Practical efforts of climate adaptation often call for the localization of these flows within cities, creating more circular economies and greater predictability and control of key urban inputs, such as food and energy, while reducing external ecological damage and enhancing biodiversity and ecosystem services^57^. Given that cities, as complex systems, are averse to being managed and planned as engineering systems^58^, how can we identify and promote effective urban management and planning in the context of adaptation to the effects of climate change?

### The social construction of urban climates

In the Anthropocene, urban climates are partly shaped by human actions and socioeconomic processes. Urban areas are modified for specific land uses and such changes in land-cover patterns in turn affect local and regional scale climates^59^. One such example is the urban heat island effect, which exacerbates socioeconomic disparity given that it disproportionately impacts poor and minority communities^60^. The effects of climate change are themselves modulated by social arrangements: for instance, the ability of urban communities to deal with altered water supplies, due to frequent and more intense droughts, is greatly determined by socioeconomic status^61^.

Trade-offs in urban adaptation are associated with different pathways for managing urban expansion. Climate change science has not yet adequately incorporated insights from urban spatial sciences and place-based studies of human-climate interactions^62^. Consequently, city policymakers and urban planners rarely use climate model data resolved at neighborhood or even city scales. Understanding how land use policy decisions shape urban climate and its impact on social equity is critical for urban communities to adapt to climate change. At the same time, climate change adaptation raises questions of data justice, in terms of both what data is available and who has access to the relevant data.

Increasing recognition of the important role urban scientists play in implementing climate solutions provides an opportunity to redesign climate models and decision-support tools to meet the needs of communities. New research initiatives increase the spatial and temporal resolution of climate model predictions and foster collaboration with vulnerable local communities to make sense of physical indicators through the lens of their living experience and their own adaptation challenges^63–65^. As climate models become more finely resolved and accurate, they can provide information relevant to neighborhood-level adaptation. This includes, for example, the quantification of risk, frequency and magnitude of heat or flooding events, and the expected consequences of adaptive responses (including inaction) in diverse local urban communities.

The growing integration of physical climate predictions with the heterogeneous and dynamic environments of urban agglomerations raises critical questions for urban adaptation research. How does local geophysical information flow through a city’s social network to influence risk perceptions and collective action? What insights on urban adaptation can be gleaned from research on information processing and collective computation and how can these be operationalized? How does the interplay of socioeconomic dynamics and urban form modulate urban microclimates? How does climate change affect the biodiversity-ecosystem services-urban ecology nexus? Answers to these questions must harness the transdisciplinary, networked character of urban adaptation research and practice as an integrated social, environmental, and technological problem. These linkages are only now becoming actionable, bringing into play feedback between climate change, engineering and political decisions in local contexts aimed at specific objectives. Recognizing that humans partly construct urban climates leads to a recognition that cities are embedded in natural systems―nature is not an externality.

### Governance and collective action for urban adaptation

Urban adaptation poses new challenges for governance and requires novel coordination arrangements with greater agility and scope to face the great unknowns and uncertainties. It also requires removing the institutional obstacles that have historically undermined the implementation of mitigation measures. Urban areas, including associated governance structures, are part of regional systems comprising interdependent urban and non-urban communities^66,67^. The emergence of urban megaregions redefines institutional boundaries and the form of governance. The distinction between urban and rural varies from country to country. In general, cities and their systems, proximal and distant, are interdependent through infrastructure, migration channels, and trade networks^68^. These systems are also connected locally and globally by ecosystem processes as well as natural and built infrastructures, which influence and are influenced in turn by human behavior. Urban adaptation research must therefore grapple with interdependencies among the three major actors (public, private, and citizens) and the challenges of governance and collective action they generate^69^.

While cities are increasingly connected in global networks of finance, knowledge, and institutional arrangements, the strategies and models of urban adaptation planning will necessarily need to recognize the unique socio-cultural histories, political realities, and geographies of individual urban places. Understanding climate policy adoption and the opportunities for multi-level collective action to advance climate justice within a greater diversity of urban socio-political contexts is critical. Governing urban systems and regions for resilience and adaptation involves diverse actors and organizations with overlapping administrative mandates and scopes of influence^70^ and actors with differing understandings, worldviews, and narratives of climate change^71^. Urban actors have varying degrees of agency and power and have different and convergent interests and variable temporal visions. Urban climate shocks underscore current limitations in institutional arrangements. Innovations such as city resilience or sustainability programs face challenges and trade-offs in practice, particularly when they take limited stock of existing efforts^72^. Developing a shared vision among the different actors is a critical starting point to create transformative change; this effort requires explicit recognition of roles and responsibilities and acknowledges that conflict is often a signal of deeper rifts in values, knowledge and power^73^.

Current inequalities in resource access, risk exposure, and participation in urban decision-making often lead privileged classes to attempt to insulate themselves from climate risk. While climate-gating^74^ may work in the short run, the reality of urban connectivity undermines such protectionism in the long run. Increasingly, citizens are part of constellations of differently managed public and private spaces in cities, such as gated communities, special improvement districts, private developments and industrial or retail spaces. These may be governed based on stakeholder goals (e.g., profit) with different implications for achieving public policy goals^75^. Mitigating or adapting to climate change are rarely among the stated goals. Recognizing diversity as an urban asset, conflict and contestation show where attention is needed to address the roots of injustice based on class and power^76^. Work in urban sociology shows how neighborhood inequalities threaten governance of environmental and social sustainability^77,78^.

### The challenge and opportunity of informality

Precarious structures of habitation and of employment opportunities, often referred to as ‘informality’, are widespread in rapidly urbanizing areas, particularly in the peri-urban locations of South Asia, Africa and South and Central America. While informality is not exclusively associated with the urban poor, in much of the world urban informality is often associated with a lack of public services, inadequate housing and high-risk exposures to health and environmental hazards. Rather than a given outcome that can be confined to a restricted or bounded area, informality is better understood as a set of overlapping processes that influence people’s urban experience and generate overlapping inequities such as lack of access to electricity, water sanitation, education, and other public goods and services, and that may occur across the city^79^.

We view urban informality as “an organizing logic, a system of norms that governs the process of urban transformation itself”^80^: (p. 148). We also note that ‘slums’ are communities that themselves exhibit differentiated levels of wealth and access to resources among the households that constitute them.

Worldwide, a third or more of the urban population lives in informal settlements or slums^81^. While being heterogeneous enough to resist sweeping characterization, informality implies the lack of recognition by formal governing authorities of land tenure arrangements and property rights, leading to a lack of legal protections, and formal supporting regulation of economic activities and settlements^82^. Informal housing for the poor—in the form of slum and squatter settlements—is built by communities themselves, in the absence of formal planning and regulations. The absence of these creates uncertainty for residents and city governments and discourages long-term investment and formal adaptation strategies that could enhance resilience and create pathways out of poverty^83^.

The urban poor in the Global South are among those populations most at risk from climate change and associated extreme weather events. A specifically critical vulnerability is the lack of adequate housing and access to services and employment. This problem is present in different cities in distinct forms: in high-growth wealthy urban areas, it often appears in the form of homelessness or inadequate housing, leading to a lack of cooling on hot days or flood protection. Historically, in all fast-growing cities of the past and present, this problem also presents itself as informal settlements, which typically lack basic services (e.g., digital divide), thereby compounding socioeconomic and civic deprivation.

Climate change has confounded development challenges, but it has also created new opportunities for strategic combinations of development and climate adaptation policies to leverage their mutual complementarities and co-benefits^84^. While slums may in some cases act as poverty traps^85^, socioeconomic opportunities and services are often better in urban slums than in rural areas of developing nations^86^. Several urban planning innovations already point the way to new kinds of climate adaptation knowledge and practice^87^. Often, informal communities themselves develop innovative responses to climate change impacts^88,89^. However, bearing the burden of adaptation can exacerbate chronic poverty. Moreover, communities alone cannot be tasked with building adaptive capacity^90^.

The availability of data for the study of urban informal communities is much improved now compared to a few years ago. In response to the lack of official data about marginalized communities and informal settlements, described by UN-Habitat in 2003 as a crisis of information^91^, a new movement was born bringing together NGOs, local community organizations, international researchers, and technologists to create appropriate methods and tools for assembling datasets about the various communities residing in slums^92^. These data collections―such as the Slum Dwellers International’s (SDI) ‘know your city’ campaign^93^ or UN-Habitat’s Urban Observatories^94^―show how comparable and verifiable data can be collected in tens of thousands of informal settlements that reflect the lived experience and priorities of local communities, while also addressing the needs of local governments and international organizations. At the same time, efforts to collect this data confront the same inequalities in knowledge production inherent to the scientific process. Residents of informal settlements often faced a deficit of credibility in putting forward their experiences and knowledge. Multiple efforts to collect data lead to research fatigue, especially when those efforts do not result in tangible improvements in the quality of life of urban residents. The challenge remains for various sectors to develop inclusive and actionable innovation to mitigate climate impacts on these communities and to address the epistemic injustices that they face^95^.

Community data collection efforts can bring together different stakeholders around agreed social and spatial facts^96,97^. These efforts facilitate collaborative design to improve well-being in urban informal communities. Crucially, they show local lived experience in ways that can drive coupled development and climate adaptation policy. Such local knowledge from the disadvantaged can act as a corrective force during political debate and implementation and drive visions and innovations. In addition to community-driven data collection, many other organizational and technological developments are radically changing what is possible to know and do, with increasing sensitivity and precision, all around the world^98^. Collaborative mapping (e.g., via OpenStreetMap) has expanded the realm of geospatial information relevant to urban (climate) adaptation, while high-precision remote sensing, coupled with Artificial Intelligence and data science methods, allows us to identify functional elements of urban environments (e.g., buildings, utilities, roads, trees, drainage) with greater spatial detail than was previously possible. These new tools and approaches are helping to establish the groundwork for a better understanding of comparative development patterns in cities.

Local community organizations are already distilling their experiences, knowledge, and expertise for addressing climate risk and exposure and engaging in dialogue with funders, governments and NGOs^99^. The urban science and climate change research communities need to join these discussions to learn from the many ways poor urban communities have implemented adaptation for years now. These efforts cannot be naïve, however, to the fact that communities can be justifiably weary of outsiders using their data, of the politicized settings in which such data is collected and that community data collection efforts are occurring in a context in which urban data is being turned into a valuable commodity by consulting firms.

### Historical processes and temporal horizons of adaptation

Discussions about adaptation to climate change convey an urgency that implies short timelines. Cities are already experiencing impacts, and policy responses tend to be dominated by short-term concerns and trends. As rapid urbanization proceeds, cities continue to grow in population and expand in areal extent, and extreme weather events are becoming more frequent. Despite the time compression posed by current trends, we propose that expanding the historical scope of urban adaptation research is critical to reveal the large-scale patterns in the interactions between nature and society that ultimately shape nuanced prospects for adaptation^100^. Research into urban adaptation is necessarily a science of place-based histories, interweaving people, human and natural landscapes, and institutions. It is also a scientific endeavor that recognizes the historical inequalities embedded in urban forms and in the process of knowledge production, and the need to incorporate a wide range of experiences to understand urban adaptation.

The historical record teaches us that communities can adapt to climate change and exogenous shocks. Worldwide urbanization has occurred, uninterrupted, for the past 7000 years. In this span, individual settlements have come and gone, and some urban systems previously representing prosperous and flourishing societies have vanished; yet many cities and urban systems have lasted for hundred and even thousands of years^101^. To achieve such endurance, problems had to be recognized, solutions devised, collective action coordinated, institutions, norms and beliefs adjusted, new technologies deployed, and previously adequate ways of doing things modified or abandoned. Studying how urban societies and communities have survived, adapted, and thrived over long periods may reveal the properties of resilient urban systems that enable them to confront different types of changes successfully. The urban past is crucial for developing a theoretically rich and empirically robust understanding of cities and urbanization^102^. Studying the history of urbanization brings a broader range of human experiences and cultures to the development of a robust understanding of urban adaptation^103^.

Heritage sites, and their study, can be a source of understanding how current forms of living have come to be and, for contemporary communities, a source of creativity in deciding what elements of heritage matter to them and which they want and hope to carry forward with them into the future^104^. The past is not, however, a source of clear, ready-made answers, nor is it destiny. The history of past urban adaptations creates the choice sets available to urban actors today and these reflect the values, priorities, power relations and consequent actions of urban residents who lived decades, and sometimes centuries, before the present. A long-term, intergenerational perspective is needed to bridge the past with the future with sufficient scope and make visible the structural injustices that must be addressed for building more sustainable, and thus adaptable, urban futures^105^.

### A convergent research agenda for urban adaptation

Developing a new research effort on urban adaptation consistent with the focus areas and tenets outlined above calls for constructing a convergent and open research program. Climate change adaptation is a specific and compelling scientific problem, inspired by pressing societal challenges, requiring deep integration across disciplines, and the construction of new analytical frameworks^106^. It is a problem that demands a multi-perspective approach, such as the one developed in the IPCC, engaging with concrete, place-based challenges. Such an effort will require changes in how relevant communities engage in research and collaborate with one another. This class of problem-oriented, convergent, research has transformed the production of scientific knowledge on urgent societal problems, as demonstrated by the global network of COVID-19 researchers.

In the urban adaptation space, convergent research needs methodological innovation to address the challenges of spatial scope and temporal scale, informality, socio-political complexity, and the rapid dynamics of urban change. It also requires much greater sensitivity to the diversity of actors vested in urban systems, with their respective histories, identities and accumulated insights, and collaborative principles based on transparency, equity, and access. Co-production of knowledge needs to be more than a slogan or an aspiration^107,108^. Critically, it also requires an international commitment to research funding to enable learning and collaboration across and among Global North and Global South urban systems, supporting knowledge exchange across a far greater diversity of scientific and empirical experiences. There need to be new mechanisms to fund research co-led by disadvantaged communities in cities, working with researchers as partners rather than as objects of study; research that challenges the drivers of inequality and vulnerability; research in which there is a recognition of multiple perspectives and rights of knowledge holders, especially those who have lived through specific experiences of violence and dispossession; research that enables discussion across disciplinary orientations seeking to reach temporary consensus across a diversity of perspectives and inform action on the ground.

A research program is animated, and justified, by the questions it poses. We bring our argumentation to a close by presenting a set of questions that illustrate the sort of inquiry we are advocating for (Table 1). The posed questions are deliberately formulated to be relevant to cities and urban systems throughout the world. The themes of the posed questions correspond to and expand the adaptation processes and approaches listed in the outer ring in Fig. 1. The questions are intended to motivate a discussion on the knowledge gaps that should be addressed with an urgency matching the urgency of responding to the already present effects of climate change in urban areas. The specific answers generated by investigating the questions in specific regions and contexts should, in effect, constitute an actionable and context-relevant research endeavor. We highlight the importance of how the questions are addressed in search of answers: concerns for epistemological adequacy and response effectiveness impel us to devise co-production strategies involving both producers and users of the research outcomes in all phases of the research (in the design, implementation, evaluation, and dissemination of the research impacts). Collaboration with boundary organizations at the urban and neighborhood scales, and with marginalized communities, is critical to ensure research is transdisciplinary, locally accountable, and salient.

Urban adaptation is an emergent property of the interactions among multiple decision-makers, socioeconomic, ecological, and climatological processes, and cultural-political relations with long contested histories^70^. It involves spatial and temporal “spillovers”, risk burden and benefit externalities extending (far) beyond urban administrative boundaries, with ethical as well as material implications far into the future. The framing of what constitutes urban adaptation is critical, with significant implications for recognition, distribution, and procedural justice in the design and implementation of adaptation solutions.
